# Characteristics of Biodegradable Gelatin Methacrylate Hydrogel Designed to Improve Osteoinduction and Effect of Additional Binding of Tannic Acid on Hydrogel

**DOI:** 10.3390/polym13152535

**Published:** 2021-07-31

**Authors:** Ji-Bong Choi, Yu-Kyoung Kim, Seon-Mi Byeon, Jung-Eun Park, Tae-Sung Bae, Yong-Seok Jang, Min-Ho Lee

**Affiliations:** 1 Department of Dental Biomaterials, Institute of Biodegradable Materials, School of Dentistry, Jeonbuk National University, Jeonju-si 54896, Jeollabuk-do, Korea; submissi@naver.com (J.-B.C.); yk0830@naver.com (Y.-K.K.); pje312@naver.com (J.-E.P.); bts@jbnu.ac.kr (T.-S.B.); 2 Dental Clinic of Ebarun, Suncheon-si 57999, Jeollanam-do, Korea; sumse1205@naver.com

**Keywords:** gelatin methacryloyl, osteoinduction, tannic acid, crosslinking, hydrogel, biodegradable

## Abstract

In this study, a hydrogel using single and double crosslinking was prepared using GelMA, a natural polymer, and the effect was evaluated when the double crosslinked hydrogel and tannic acid were treated. The resulting hydrogel was subjected to physicochemical property evaluation, biocompatibility evaluation, and animal testing. The free radicals generated through APS/TEMED have a scaffold form with a porous structure in the hydrogel, and have a more stable structure through photo crosslinking. The double crosslinked hydrogel had improved mechanical strength and better results in cell compatibility tests than the single crosslinked group. Moreover, in the hydrogel transplanted into the femur of a rat, the double crosslinked group showed an osteoinductive response due to the attachment of bone minerals after 4 and 8 weeks, but the single crosslinked group did not show an osteoinductive response due to rapid degradation. Treatment with a high concentration of tannic acid showed significantly improved mechanical strength through H-bonding. However, cell adhesion and proliferation were limited compared to the untreated group due to the limitation of water absorption capacity, and no osteoinduction reaction was observed. As a result, it was confirmed that the treatment of high-concentration tannic acid significantly improved mechanical strength, but it was not a suitable method for improving bone induction due to the limitation of water absorption.

## 1. Introduction

Bone defects are health-threatening diseases and are caused by various factors such as trauma, genetics, and cancer. The number of patients increases with age. Although bones can be regenerated, the ability widely varies from person to person. Currently, the most common method to recover the damaged bone defects is the direct implantation of a bone-grafted material into the defective area. Bone grafting must include essential elements of bone regeneration, namely osteoinduction, osteoconduction, and osteogenesis, in conjunction with the final bonding between the bone and the graft material [1]. In bone tissue engineering, various complex processes involving cell adhesion, migration, proliferation, differentiation, and matrix formation are used while applying biomaterials to induce bone generation [2]. To recover functions, often, biomaterials containing bioactive substances are used [3]. Hydrogels made of natural and synthetic biomaterials that similarly mimic the structure and biological properties of the natural extracellular matrix have long been studied as candidates for cell delivery in medicine and dentistry [4].

Gelatin is a type of derived protein partially extracted from collagen by thermal or chemical denaturation. It is suitable for hydrogels due to its retentive ability for a motif of peptides that are degraded by matrix metalloproteinase (MMP) and arginine-glycine-aspartic acid (RGD) related to cell adhesion. Additionally, gelatin has an excellent biocompatibility and swelling ratio [5,6,7,8]. However, gelatin dissolves in water at a body temperature above 37 °C and does not offer structural stability. Its physical properties are improved by chemical crosslinking with glutaraldehyde or 1-ethyl-3-(3-dimethylaminopropyl)-carbodiimide) (EDC)/(N-hydroxysuccinimide) (NHS) [9,10]. However due to crosslinker toxicity, many restrictions were imposed as referred from previous studies [11,12,13].

The recently developed gelatin methacryloyl (GelMA) can be produced by chemical modification of the amine and hydroxyl groups of gelatins, through which the hydrogel can be covalently crosslinked in the presence of photoinitiators and light [14]. In addition, GelMA hydrogels irreversibly change some structures due to hydrolysis and chemical modification but retain some properties of collagen and gelatin, such as cell adhesion, heat sensitivity, and enzymatic degradation [6,14,15]. GelMA hydrogels support the formation of novel ECMs, are enzymatically degradable, can be produced at low cost, are readily crosslinked under physiological conditions, and show potential for tissue engineering [16].

Irgacure 2959 (2-hydroxy-4′-(2-hydroxyethoxy)-2-methylpropiophenone) is the most commonly used in tissue engineering applications [17,18,19,20]. However, its low water solubility and UV light (365 nm) exposure cause potentially harmful effects on cells and tissues. Prolonged exposure to UV light may damage the DNA and cellular functions [21,22,23]. However, visible light (VL) uses a longer wavelength (405 nm) and penetrates further into the tissue during treatments. Additionally, no heat is generated, and cell damage is minimal [24]. The biocompatibility of GelMA hydrogel in bone tissue engineering has been demonstrated by many studies [25,26,27,28,29,30]. However, hydrogels fabricated with GelMA have lower mechanical strength than other natural and synthetic polymeric hydrogels [31,32,33].

Recently, hydrogels made with double crosslinking (DC) are attracting a lot of attention due to their excellent mechanical performance [34]. In addition, methods have been proposed to adjust the mechanical properties of GelMA using several crosslinking steps. For example, Rizwan et. al. achieved double crosslinking by performing physical crosslinking and then photo crosslinking [35]. In another study, Zhou et. al. used enzyme crosslinking followed by photo crosslinking to improve the viscosity of GelMA for bioprinting [36].

Tannic acid (TA) is a natural polyphenol compound with biological antioxidant and antibiotic properties [37]. However, TA forms an amorphous structure with a complex coagulation behavior in hydrogels making it difficult to control these strong interactions [38,39]. Accordingly, macromolecules such as DNA proteins are used to balance the change [40], or multiple steps are applied on TA under controlled conditions [41]. Similar to polydopamine inspired by mussels, a high pyrogallol and catechol content of the TA molecular structure can improve compressive and tensile properties via bonding [42].

**Figure 1:**
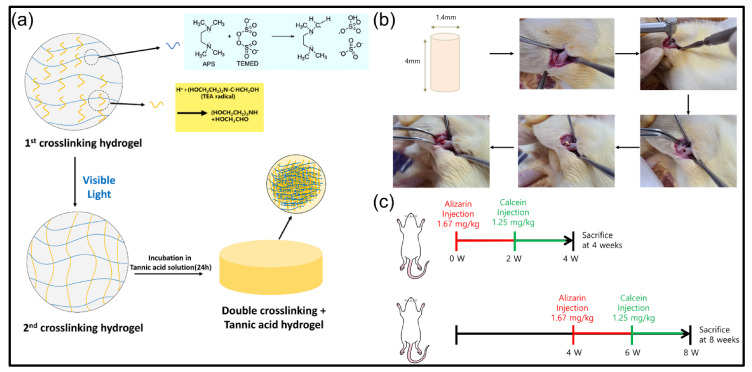
Schematic diagram of (**a**) Fabrication of double crosslinked hydrogel and tannic acid treatment, (**b**) Sample type and location for in vivo test (Sample placement in the femur), (**c**) Group division according to the schedule of staining reagent injection time for checking and analyzing bone formation behavior.

In this study, based on the excellent binding ability of the GelMA hydrogel fabricated using double crosslinking and TA (see Figure 1), it was evaluated through analysis whether the hydrogel network could be strengthened in a well-arranged manner by TA. In addition, changes in mechanical properties were observed when the double crosslinked hydrogel and TA were applied to the hydrogel, and the effect on biodegradation and osteoinduction when finally implanted in an animal model was observed. Based on this, it was attempted to confirm whether the manufacturing method using double crosslinking showed better effects than the manufacturing method using single crosslinking. In addition, we tried to determine whether the improvement of mechanical strength when TA was combined and whether TA had an effect on the improvement of bone induction.

## 2. Materials and Methods

All materials used in this experiment, including type A gelatin, tetramethyl ethylene diamine (TEMED), ammonium persulfate (APS), methacrylic anhydride (MA), and tannic acid (TA), were purchased from Sigma Aldrich (Yongin, Korea). All chemicals were used without further purification.

### 2.1. Systhesis of Gelatin Methacryloyl

According to a previously reported study [43], methacryloyl-bonded GelMA macromonomer was synthesized. A total of 10% (*w*/*v*) of gelatin was completely dissolved in Dulbecco’s phosphate-buffered saline (DPBS) at 60 °C and magnetically stirred. Then, 8 mL of MA were added to the gelatin solution and stirred at 50 °C for 2 h. The GelMA solution was then diluted in pre-made DPBS to increase the volume by 5 times and terminate the reaction. The GelMA solution was dialyzed against deionized water for 1 week at 40 °C in a 12–14 kDA cutoff tube. Subsequently, the solution was freeze-dried for 5 days, and the resulting GelMA was stored in a −20 °C freezer until further use.

### 2.2. Fabrication of Single and Double Crosslinking Hydrogels

The concentration of GelMA solution was fixed at 15% based on previous experi-mental results [44]. The prepared GelMA foam was dissolved in deionized water at 50 °C for 2 h. The hydrogel prepared by single crosslinking was prepared using low-temperature crosslinking and light crosslinking.

A hydrogel using low-temperature crosslinking was prepared by dissolving 14% (*w*/*v*) ammonium persulfate (APS) and 7% (*w*/*v*) tetramethylethylenediamine (TEMED). This prepolymer solution was pipetted into cylindrical (1.5 mm diameter, 1 mm thickness) polystyrene molds and placed in a freezer set to −20 °C. Low-temperature crosslinking was allowed to proceed for 18 h, and the resulting hydrogel was thawed and hydrated in dH_2_O prior to use.

For photo crosslinking, 1.88 (*v*/*v*) triethanolamine (TEA), 1.25 (*w*/*v*) Vinylcaprolactam (VC), and 0.5 mM Eosin Y disodium salt were sequentially added to the prepared 15% (*w*/*t*) GelMA solution and mixed, and then the prepolymer solution was pipetted into a cylindrical (1.5 mm diameter, 1 mm thick) polystyrene mold and exposed to visible light for 120 s.

For double crosslinking, 14% (*w*/*v*) ammonium persulfate (APS) and 7% (*w*/*v*) tetra-methylethylenediamine (TEMED) were sequentially added to the prepolymer solution used for photo crosslinking and mixed. Then, low-temperature crosslinking was per-formed in a freezer set at −20 °C. for 18 h, and then exposed to visible light for 120 s before thawing to form a double crosslinked hydrogel.

### 2.3. Fabrication of Hydrogel Applied with TA

The fabricated hydrogel was immersed in the tannic acid (TA) solution of previously pre-pared concentrations (10%, 50%, and 100% (*w*/*v*)), and shaken for 24 h on a shaker. Then, the hydrogel was washed three times with deionized water to remove excess TA. Hydrogels used for the experiment were fabricated with a 10 mm diameter and a 3 mm height and were referred to as GelMA-S (low-temperature crosslinking), GelMA-V (photo crosslinking), and GelMA-D (double crosslinking). Depending on the concentration of TA, additional indicators, T10, T50, and T100, were used.

### 2.4. Fourier Transform Infrared Spectroscopy (FT-IR) Characterization

FT-IR analysis was used to investigate the intermolecular interactions between TA, double crosslinking, and the GelMA Spectra that were obtained at room temperature using a FT-IR spectrometer (Perkin Elmer, Waltham, MA, USA). FT-IR analysis was performed within the wavelength 4000–500 cm^−1^ (KBr) using the attenuated total reflectance (ATR) method.

### 2.5. Mechanical Tests

Mechanical properties of the hydrogel were measured using a universal testing machine (Instron 5569, Instron, Norwood, MA, USA). All the first compression tests were performed on a cylindrical hydrogel at a 2 mm/min rate with up to 95% maximum load of a 50 N load cell. After the first compression test, damaged samples were eliminated, and the second compression test was conducted at a 0.5 mm/min rate and up to a 95% maximum load of a 500 N load cell. The second compression test data were automatically calculated using the Bluehill 2 software. The compressive modulus was calculated as the slope of the linear region (0–20%) of the stress-strain curve. All samples were hydrated during the test.

### 2.6. Swelling Ratio Tests

The prepared GelMA hydrogel was incubated at 37 °C for 24 h. After the sample was removed from the solution and the residual liquid separated using Kimwipe, the weight, W_s_, was measured. The weight of the freeze-dried hydrogel was measured as W_d_. The swelling ratio was calculated according to Equation (1).

Swelling Ratio: SR = (W_s_ − W_d_)/W_d_ (1)

### 2.7. FE-SEM Characterization

The freeze-dried hydrogel was placed on a wafer for platinum coating. The shape of the hydrogel was observed using a FE-SEM (Hitachi, Tokyo, Japan).

### 2.8. Evaluation of In-Vitro Cell Proliferation

Osteoblast cells were used in this study, MC3T3-E1 (ATCC; American Type Culture Collection), to evaluate their effect on bone formation. For the culture medium, a nutrient component, 10% fetal bovine serum (Gibco Co., Waltham, MA, USA) containing an antibiotic (penicillin), was added to an α-MEM (Gibco Co., Waltham, MA, USA) medium. The cell culture was conducted in an incubator (Thermo Electron Corporation, Waltham, MA, USA) in a 5% CO_2_ atmosphere at 37 °C. A water soluble tetrazolium (WST) assay was used to evaluate cell proliferation by placing samples in a 48-well plate and incubating the MC3T3-E1 cells with a cell density of 1 × 10^4^ cells mL^−1^ for 1, 3, and 7 days. Then, the medium was removed, replaced with 400 µL of CCK-8 (Enzo Life Science Inc., Farmingdale, NY, USA) reagent mixed with α-MEM medium, and stored in the incubator with 5% of CO_2_. After 90 min, 100 µL were added to a 96-well plate, and the absorbance was measured at 450 nm using the ELISA reader (Molecular devices, Silicon Valley, CA, USA).

### 2.9. Evaluation of Bone Regeneration and Mineral Activity In Vivo

The effect of bone remodeling was compared to the rat femur defect model applying GelMA-P, GelMA-PT100, GelMA-VP, and GelMA-VPT100 groups (*n* = 3). Experiments in this study were conducted under the protocols approved by the Institutional Animal Care and Use Committee of the Chonbuk National University Laboratory Animal Center (CBNU 2020-094) following the declaration of Helsinki. The prepared freeze-dried hydrogel samples were implanted on the proximal femur from the outer side for each rat. Male Sprague–Dawley rats (*n* = 16), used in this experiment, were about 8 weeks old with an average weight of 280 g. The rats were purchased from Damul Science (Daejeon, Korea) and used after a week’s adjusting period. For anesthesia, 0.6 mL/kg tiletamine and zolazepam (Zoletil 50, Virbac Laboratories, Carros, France) and 0.4 mL/kg xylazine hydrochloride (Rompun, Bayer Korea, Seoul, Korea) were intramuscularly injected into the leg of each rat. The surgical site of the anesthetized rat was shaved, and an approximately 1 cm incision was made on the femur after sterilization using povidone iodine. After raising the flap due to the incision, a contra-angle handpiece (X-smart Endodontic Motor, Dentsply Maillefer, Switzerland) equipped with a 1.6 mm pilot round head bur (H1.31-0.16, Lemgo, Germany) was used to create a hole in the cortical bone. After the surgery, an antibiotic (Amikacin, Samu Median Co., Ltd., Seoul, Korea) was subcutaneously injected (0.6 mL/kg). At 4 and 8 weeks after implanting the hydrogels, the rats were sacrificed to obtain the femoral bone blocks containing the sample.

#### Fluorescence Staining

Alizarin complexone (red) and calcein (green) fluorescent materials were used to observe the mineralized bones. To evaluate a bone-forming ability on the samples after 4 and 8 weeks, Alizarin complexone (red) solution (1.67 mL/ kg, body weight) was injected at 0 and 4 weeks, and calcein (1.25 mL/kg, body weight) was injected into the peritoneum at week 2 and week 6. After 4 and 8 weeks, the femoral bone blocks were obtained from the sacrificed rats and were fixed in 10% formaldehyde solution to fabricate a resin-embedded tissue slide. Then, all blocks were dehydrated using an increased concentration of ethanol, and methyl methacrylate (MMA, JUSEI Chemical Co. Ltd., Tokyo, Japan) was inserted into the bone. The bone blocks with penetrated MMA were embedded in an activated MMA resin. The embedded blocks in the resin were sectioned along the longitudinal axis of the embedded sample. The fluorescent-stained tissue slide of the sectioned sample was observed using a Super Resolution Confocal Laser Scanning Microscope (Carl Zeiss AG, Oberkochen, Germany). Histological images of bone staining with alizarin complexone (red) and calcein (green) were obtained at 543 nm and 488 nm, respectively.

### 2.10. Statistical Processing

To evaluate the difference among groups, SPSS ver 21.0 (SPSS Inc., Chicago, IL, USA) software was used. One-way ANOVA was used to assess three or more groups within one factor, and Tukey’s postmortem analysis was used to evaluate the average. In all experiments, if the *p*-value was < 0.05, it would determine significant differences in the groups.

## 3. Results and Discussion

### 3.1. Formation of GelMA and TA Treatment of GelMA Hydrogel

**Figure 2:**
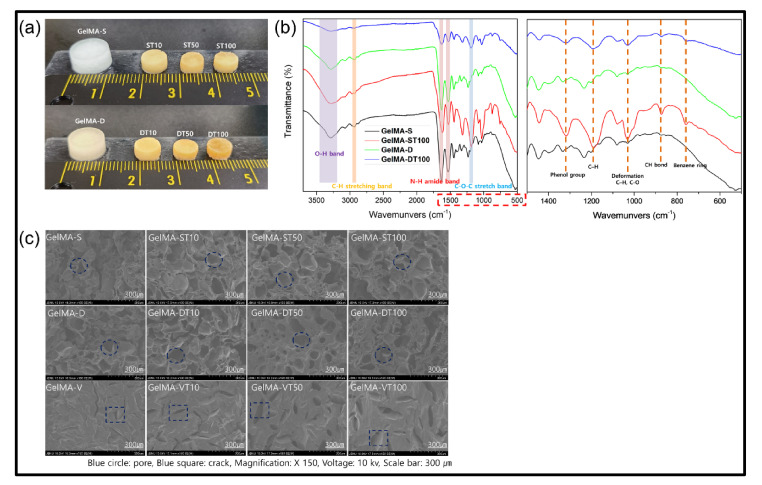
(**a**) The representative morphological changes from pristine GelMA hydrogels (left) to GelMA-TA (right) after a 24 h incubation in TA solution. Concentrations of GelMA and TA are 15% (*w*/*v*), and 10%, 50%, 100% (*w*/*v*), respectively. (**b**) FT-IR spectra of hydrogels. (**c**) FE-SEM of GelMA hydrogels, and GelMA-TA hydrogel 10%, 50%, 100% TA, 24 h treatment time. Red circles indicate pores, and squares indicate cracks. The image was measured using a voltage of 10 kv, the magnification was 150×, and the scale bar was 300 µm.

We prepared a hydrogel synthesized by single crosslinking and double crosslinking according to the plan, and TA solutions with different concentrations were applied. As shown in Figure 2a, it can be seen that GelMA-D has changed color under the influence of Eosin Y. In addition, GelMA (15% *w*/*v*) hydrogel (cylindrical height 2 mm, diameter 10 mm) changed to opaque with a decrease in size after 24 h treatment of TA (10%, 50%, 100% *w*/*v*), which shows a real interaction between GelMA and TA.

To confirm the interaction further, the Fourier Transform Infrared (FT-IR) study was performed (Figure 2b). Spectra from the remaining groups except for GelMA-S generally show that high transmittance at about 3100–3600 cm^−1^ is closely related to hydrogen bonding, which is due to the shift of O-H groups by additional bonding [45,46]. In addition, the peaks of the amide groups (I, II, III) characteristic of GelMA hydrogels were observed at around 1612 cm^−1^, 1520 cm^−1^, 1436 cm^−1^ without appreciable changes in intensity and frequency. These results show that covalent bonds are not present [47]. The peak at 1319 cm^−1^ is caused by the phenol group of TA. The peak at 1198 cm^−1^ is due to C-H, and the vibration peak at 1100–1000 cm^−1^ is due to C-O and C-H deformation. The peak in 550–900 cm^−1^, which is characteristic of TA, is based on the C-H bond of the benzene ring [48].

Morphological analysis of the prepared GelMA and TA treatment of GelMA hydrogels was measured by FE-SEM (Figure 2c). For comparison, a hydrogel (GelMA-V) formed by photo crosslinking was additionally observed. GelMA-V showed agglomerated surfaces and partial cracks due to polymerization by radicals. The hydrogel formed by low-temperature crosslinking (GelMA-S) had a porous microstructure as the ice crystals formed by the APS/TEMED reaction were removed. The hydrogel formed by double crosslinking (GelMA-D) was photo crosslinked after low-temperature crosslinking and showed a similar shape to GelMA-S. Although an increase in pore size was observed with increasing TA concentration (10%, 50%, and 100%) in GelMA-V, the size did not increase noticeably after 50% concentration. In GelMA-S, it was confirmed that the wall of the porous structure was slightly thickened by the binding of TA, and in GelMA-D, more TA binding than GelMA-P was observed. However, looking at the overall trend, no significant difference in surface shape was observed between GelMA-S and GelMA-D groups.

### 3.2. Mechanical Properties of Htdrogel

**Figure 3:**
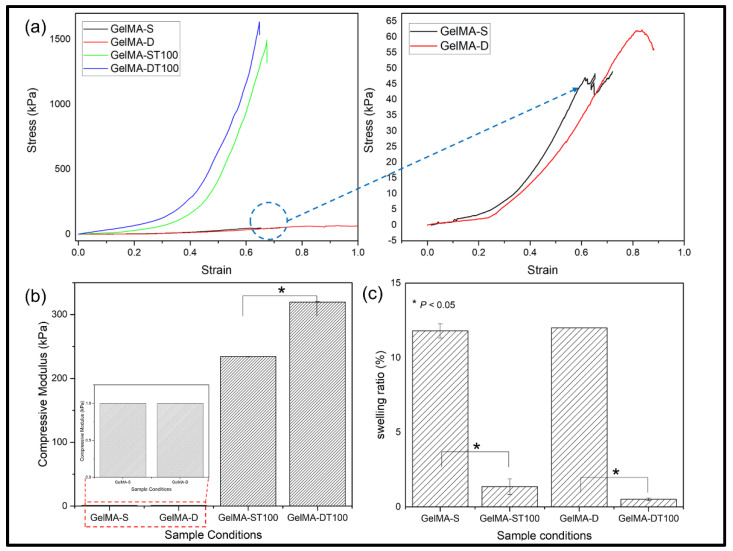
Mechanical properties of hydrogels (GelMA-S and GelMA-D) and after treated with the 100 *w*/*v*% of TA. The compression deformation of all hydrogels was performed up to 95%; (**a**) Stress-strain Curve for hydrogels, (**b**) Compressive Modulus through (0~20% slope), (**c**) Swelling ratio of GelMA and GelMA-TA hydrogels. (Data are presented as mean ± SD, *n* = 3, * *p* < 0.05).

Figure 3a shows a typical compressive stress-strain curve. In the case of the TA-treated group, it was confirmed that the fracture stress was significantly improved. In particular, the untreated and TA-treated hydrogel groups showed the same pattern and showed stronger fracture stress in the double crosslinked group than in the single crosslinked group.

These results showed the same trend in the compressive stress (Figure 3b). The compressive stresses of each group were GelMA-S: 46 kPa; GelMA-D: 62 kPa; GelMA-ST100: 234 kPa; GelMA-DT100 showed 319 kPa, and it was confirmed that the improvement was about 1.3 times between the single crosslinked and double crosslinked groups. In addition, it was confirmed that there was a difference of about five times between the untreated group and the TA group. TA bound to the hydrogel strengthened the bond with the GelMA network through hydrogen bonding and hydrophobic force, and showed improvement in mechanical strength [49]. Mechanical properties can be affected by the water content of the hydrogel. Therefore, the GelMA-S, GelMA-D, GelMA-ST100, and GelMA-DT100 groups were immersed in PBS and then cultured in an incubator at 37 °C for 24 h.

The smoothing behavior of the hydrogel was then characterized and shown in Figure 3c. The swelling behavior of GelMA-S and GelMA-D showed similar results, and no significant difference was observed. Similarly, no significant difference was observed in the swelling behavior of GelMA-ST100 and GelMA-DT100. However, a significant difference was observed when comparing the untreated group and the TA treated group. It could be confirmed that the TA-treated group was significantly restricted in swelling behavior compared to the untreated group. The low swelling behavior of the TA-treated group showed that the interaction of H-bonds between TA and GelMA could compress the structure of the hydrogel and limit water absorption [43]. As a result, it was confirmed that the low swelling ratio of the TA-treated hydrogel group indicates a higher mechanical strength.

### 3.3. Cytocompatibility of Hydrogel

**Figure 4:**
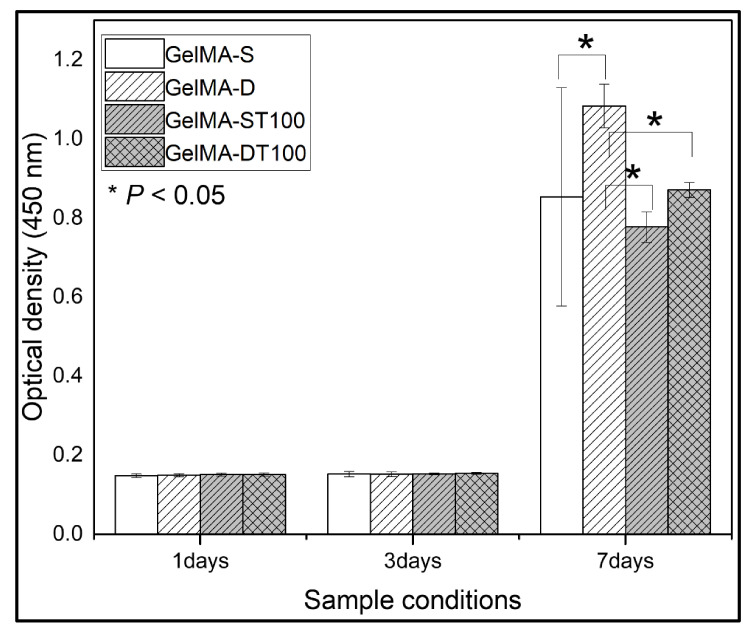
The proliferation of cells measured by CCK8 assays (WST) on days 1, 3, and 7. (Data are presented as mean ± SD, *n* = 3, * *p* < 0.05).

We investigated the cellular compatibility of GelMA and GelMA-TA hydrogels. Pro-osteoblasts (MC3T3-E1) were cultured in flat 48-well plates with or without hydrogels. According to previously known research results, the differentiation of MC3T3-E1 cells showed better cell activity in soft materials than in hard materials [50]. The single and double crosslinked hydrogels used in the study have soft matrix properties, whereas the TA-treated GelMA hydrogels have more rigid matrix properties. Our experiments also showed similar results. Cell viability was confirmed using quantitative detection of cck8 at 1, 3, and 7 days after seeding (Figure 4). UV absorbance increased with increasing incubation time for days 1–3 due to cell proliferation, but there was no statistical difference in cell viability in all groups. However, on day 7, there was a change between each group. GelMA-D showed better UV absorbance, and a significant difference occurred compared to other groups.

The group treated with TA showed lower absorbance than the existing GelMA group, but there was no significant difference except for the GelMA-D group. These results also suggested that the hydrogel can be applied to in vivo use. A high concentration of TA treatment continuously released an excess of TA in the hydrogel, and it was confirmed that the released TA affects cell activity. Although the exact mechanism of the effect of TA on cellular activity is unknown, it may be due to the presence of galloyl groups in TA. A galloyl group of tannic acid interacts with proteins through hydrophobic bonding and electrostatic and hydrophobic interactions [51]. The electrostatic interaction induced by tannic acid at 7.4 pH is relatively weaker than the hydrogen bonding and fails to stabilize proteins in their native form [52]. This interaction of tannic acid suggests the adhesion prevention of osteoblasts. Nevertheless, these results suggest that the hydrogel may be applicable for in vivo use.

### 3.4. In Vivo Osteoinduction

**Figure 5:**
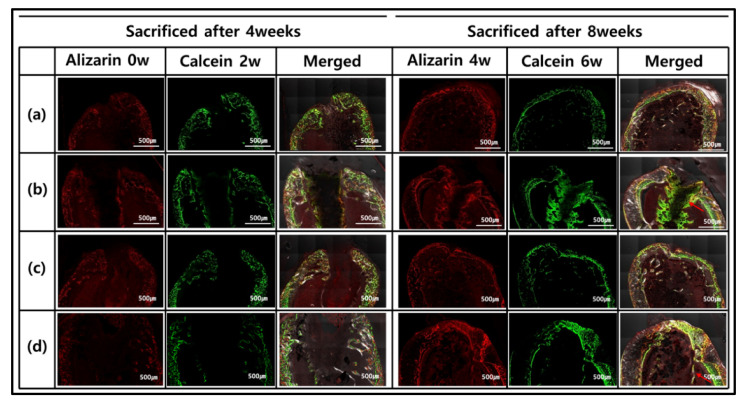
Fluorescence microscopy imaging after 4 and 8 weeks after implantation (red and green represent alizarin and calcein labeling, respectively): (**a**) GelMA-S, (**b**) GelMA-D, (**c**) GelMA-ST100, and (**d**) GelMA-DT100.

In vivo experiments were conducted with GelMA-S, GelMA-D, GelMA-ST100, and Gel-MA-DT100 groups. Osteoinduction and biodegradation were observed by implanting a hydrogel sample prepared in the form of a rod into the femur of a rat. After 4 and 8 weeks, mice were sacrificed and the results of new bone formation according to osteoinduction in the hydrogel are shown in Figure 5 through fluorescence images. Alizarin complexon (red) and calcein (green) staining was performed every 2 weeks to observe bone formation. After 4 weeks of sample transplantation, most of the staining intervals of alizarin red and calcein green were consistent, and there was no significant difference in the rate of new bone formation.

**Figure 6:**
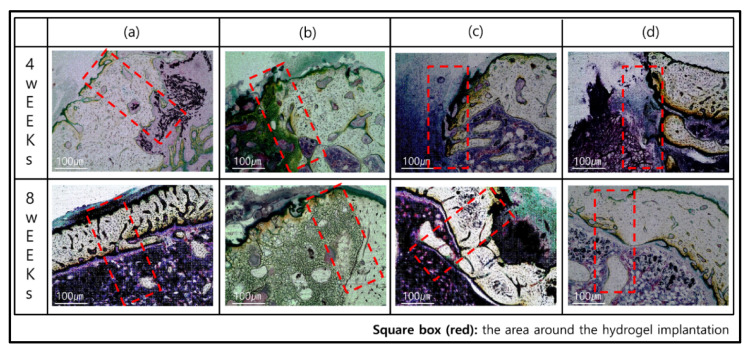
Histological images of Villanueva Osteochrome Staining for bone tissues after 4 and 8 weeks: (**a**) GelMA-S, (**b**) GelMA-D, (**c**) GelMA-ST100, and (**d**) GelMA-DT100.

However, 8 weeks after implantation, different types were observed for each group. In GelMA-S and GelMA-ST100, the hydrogel morphology and osteoinductive reaction were not confirmed. On the other hand, it was confirmed that the formation of new bone and the concentration of calcein green increased in GelMA-D and GelMA-DT100. In particular, in GelMA-D, it was confirmed that many of these minerals were generated inside the hydrogel and maintained its shape. In the process of bone remodeling, bones are synthesized by osteoblasts, and small mineral crystals are deposited between collagen molecules. Over the next few days, these crystals fill the space occupied by water, resulting in bone mineralization [53]. For hydrogel samples, it is important to have adequate strength to retain their shape and the ability to absorb moisture. GelMA-D showed that many of the main minerals were settled in the hydrogel before the hydrogel was decomposed and replaced with the degradation (Figure 5b). However, in the hydrogel treated with TA, water absorption was inhibited, and it was confirmed that the main mineral was deposited only on the outside (Figure 5d). These morphologies tend to be consistent with Villanueva Osteochrome Staining (Figure 6).

GelMA-S did not adhere to the bone 4 weeks after transplantation, and it was confirmed that rapid degradation occurred (Figure 6a). On the other hand, in GelMA-D, osseointegration was successfully achieved 4 weeks after transplantation, and it was confirmed that the external bone mineral penetrated into the sample. In addition, it was observed that the bone mineral formed in the sample was gradually converted into the new bone after 8 weeks. It is judged that the induction reaction for bone remodeling occurred smoothly only in GelMA-D (Figure 6b). After 4 weeks of implantation of GelMA-ST100 and GelMA-DT100 treated with TA, the shape of the sample was maintained, but it did not adhere to the bone and there was no osteoinductive reaction (Figure 6c,d). The location where mineral crystals are deposited between collagen fibers, one of the components of bone, and where mineralization begins, is called the hole zone [52]. This region is the first to deposit calcium and phosphorus in the inter-molecular space created when collagen molecules move out of position when forming fibers [54].

GelMA-S can be exchanged smoothly in body fluids through its porous structure, but it has a disadvantage in that rapid degradation can occur because the reaction range by the enzyme is large. Double crosslinking solved these shortcomings. The improved strength provided a favorable environment for calcium and phosphorus deposition and new bone formation before being degraded by enzymes. On the other hand, TA treatment gave high mechanical strength but did not provide a suitable environment for the formation of new bone by osteoinduction. In addition, although the exact mechanism of TA has not been elucidated, it cannot be ruled out that the adhesion of progenitor cells is hindered by the nature of TA, which does not stabilize the natural protein in the environment of pH 7.4 [55,56].

## 4. Conclusions

In this study, it was evaluated whether a hydrogel produced by double crosslinking using GelMA, a natural polymer, was suitable for improving bone induction and showed better results when additionally combined with TA.

As a result, the double crosslinked group showed improved mechanical strength and better cell compatibility than the single crosslinked group. In addition, the single crosslinked group transplanted into the rat femur showed no osteoinduction response due to rapid degradation after 4 and 8 weeks, but the double crosslinked group showed bone mineral binding and osteoinduction. The hydrogel group treated with a high concentration of TA showed a significant improvement in mechanical strength through H-bonding. However, cell adhesion and proliferation were limited compared to the untreated group, and osteoinduction was not observed in the TA-treated single and double crosslinked groups.

In conclusion, it was found that double crosslinking is a suitable method for improving the strength and bone induction of hydrogels compared to single crosslinking. Conversely, it was confirmed that the binding of high-concentration TA significantly improved mechanical strength, but delayed bone remodeling by limiting water absorption. In future research, it is expected that the development of functional hydrogels that can be used for bone tissue engineering by supporting bioactive substances or drugs on the double crosslinked hydrogel is expected.
